# Polarizable Additive with Intermediate Chelation Strength for Stable Aqueous Zinc-Ion Batteries

**DOI:** 10.1007/s40820-023-01305-0

**Published:** 2024-01-12

**Authors:** Yuting Xia, Rongao Tong, Jingxi Zhang, Mingjie Xu, Gang Shao, Hailong Wang, Yanhao Dong, Chang-An Wang

**Affiliations:** 1 State Key Lab of New Ceramics and Fine Processing, School of Materials Science and Engineering, Tsinghua University, Beijing, 100084 People’s Republic of China; 2 School of Materials Science and Engineering, Zhengzhou University, Zhengzhou, 450001 Henan People’s Republic of China

**Keywords:** Aqueous zinc-ion batteries, Electrolyte additives, DTPA-Na, Chelation strength

## Abstract

Aqueous zinc-ion batteries are promising due to inherent safety, low cost, low toxicity, and high volumetric capacity. However, issues of dendrites and side reactions between zinc metal anode and the electrolyte need to be solved for extended storage and cycle life. Here, we proposed that an electrolyte additive with an intermediate chelation strength of zinc ion—strong enough to exclude water molecules from the zinc metal-electrolyte interface and not too strong to cause a significant energy barrier for zinc ion dissociation—can benefit the electrochemical stability by suppressing hydrogen evolution reaction, overpotential growth, and dendrite formation. Penta-sodium diethylene-triaminepentaacetic acid salt was selected for such a purpose. It has a suitable chelating ability in aqueous solutions to adjust solvation sheath and can be readily polarized under electrical loading conditions to further improve the passivation. Zn||Zn symmetric cells can be stably operated over 3500 h at 1 mA cm^−2^. Zn||NH_4_V_4_O_10_ full cells with the additive show great cycling stability with 84.6% capacity retention after 500 cycles at 1 A g^−1^. Since the additive not only reduces H_2_ evolution and corrosion but also modifies Zn^2+^ diffusion and deposition, highlyreversible Zn electrodes can be achieved as verified by the experimental results. Our work offers a practical approach to the logical design of reliable electrolytes for high-performance aqueous batteries.

**Supplementary Information:**

The online version contains supplementary material available at 10.1007/s40820-023-01305-0.

## Highlights

- Design principle of a reliable electrolyte based on chelation strength is proposed for high-performance aqueous batteries.
- The addition of penta-sodium diethylene-triaminepentaacetic acid salt is effective in dynamically modulating anode/electrolyte interface, inhibiting water-related side reactions, and mitigating dendrite generation on zinc anodes.
- Symmetrical, Zn||Cu half and Zn||NH_4_V_4_O_10_ full cells using the new electrolyte exhibit improved electrochemical performance.

**Supplementary Information:**

The online version contains supplementary material available at 10.1007/s40820-023-01305-0.

## Graphical abstract

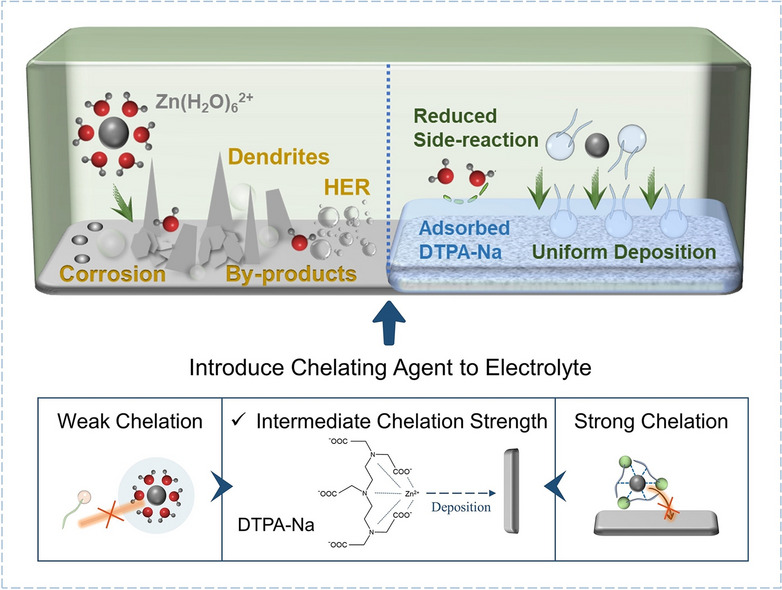

## Introduction

As renewable energy sources (e.g., solar and wind) continue to grow, the use of lithium-ion batteries (LIBs) is limited by resources and safety issues. It is urgent to search for inexpensive batteries that are based on earth-abundant elements and can safely store large amounts of energy for intermittent power generation [1–3]. Aqueous zinc-ion batteries (AZIBs) with non-flammable aqueous electrolytes have the advantages of environmental friendliness, low cost, and superior safety [4–6]. Since metallic zinc can be directly used as the anode, it has the potential for large-scale applications considering its high abundance and theoretical capacity (820 mAh g^−1^ and 5854 Ah cm^−2^) [7, 8].

Despite these unique advantages, AZIBs currently face several issues, especially poor cycling performance due to the instability of zinc metal anodes [9–12]. Because metallic zinc is not thermodynamically stable against conventional aqueous electrolytes, side reactions such as hydrogen evolution reaction (HER) and zinc corrosion are inevitable at the electrode/electrolyte interface. It lowers the reversibility and long-term stability of the cell [11, 13]. Meanwhile, Zn^2+^ tends to form hydrated zinc ions Zn(H_2_O)_6_^2+^ in aqueous solutions. It leads to strong interactions between Zn^2+^ and water molecules that set a high energy barrier for Zn^2+^ dehydration and increase charge transfer resistance. As a result, a large number of water molecules are carried to the zinc metal surface, intensifying water-related side reactions [10, 14, 15]. In addition, morphological changes in the zinc metal anode during cyclic charging/discharging process eventually result in dendrite formation, which disrupts the electrode structure and short-circuits the cell. The enlarged surface area from morphological instability may further promote undesired side reactions [16–18].

To solve the above problems in AZIB, researchers have developed modification strategies in recent years, such as artificial coatings on anode [19–22], design of 3D-structured electrodes [23–25], and novel electrolyte systems [26–32]. Among them, the electrolyte modification plays an important role in alleviating the side reactions and promoting uniform deposition of the Zn^2+^ on the anode owing to the avoiding of complicated preparation process and excessive invalid weight. For example, the highly concentrated “water-in-salt” strategy of 1 m Zn(TFSI)_2_ + 20 m LiTFSI [32] or 30 m ZnCl_2_ [33], and molecular crowding electrolytes were used to reduce the number of free water molecules in the electrolyte to suppress the activity of water, which also effectively broadens the electrochemical window of the electrolyte. However, the high cost and lowered conductivity make this strategy difficult to be applied to a large scale. On the other hand, the in-situ formation of the solid electrolyte interphases (SEIs) on the anode surface was promoted by the introduction of additives such as Zn(H_2_PO_4_)_2_ [34] and Zn(BF_4_)_2_ [35]. However, since the electrolyte additives are continuously consumed during repeated cycles due to the unstable SEIs, the long-term stability remains an issue. Some researchers have also reported that introducing additives of dimethyl sulfoxide (DMSO) [27], sodium dodecyl benzene sulfonate (SDBS) [36], and ethylene glycol [37] can replace H_2_O in the Zn^2+^-solvation sheath, thus inhibiting the side reactions related to H_2_O. In spite of this, a more rational exploration and better understanding of how to optimize the deposition process on anode surface in a long-term cycle is still needed.

From the above, one may conclude that the ideal additive should be able to effectively regulate the Zn^2+^-solvation sheath, inhibit the reaction that occurs when free water comes in contact with the anode surface, and mitigate the dendrite generation on zinc anodes. In this case, we turned our attention to penta-sodium diethylene-triaminepentaacetic acid salt (denoted as DTPA-Na), a widely-used and cheap chelating agent with strong affinity for various metal ions such as Cu^2+^ and Ca^2+^ [38, 39]. Meanwhile, analogous to the Sabatier principle about intermediate compound for catalyst design, the chelation strength with Zn^2+^ of DTPA-Na is found to be proper, which is able to adjust solvation sheath without causing significant energy barrier for zinc ion dissociation [40, 41].

We added DTPA-Na to 2 M ZnSO_4_ baseline electrolyte and explored the suitable concentration for optimal electrochemical performance. Through the strong interaction between DTPA anion and Zn^2+^, the H_2_O molecules are removed from the solvation sheath of Zn^2+^ and improve electrolyte structure. Meanwhile, because the DTPA anions have a higher adsorption capacity than the H_2_O molecules, they are easily adsorbed on the Zn surface to cover the active site of H_2_ reduction, thus not only inhibiting the side reactions such as HER but also facilitating the uniform nucleation of Zn by limiting the disordered two-dimensional diffusion [42, 43]. Taking it a step further, we proposed a logical design principle of a reliable electrolyte based on chelation strength for aqueous zinc-ion batteries. With the appropriate amount of the selected DTPA-Na additives, an ultra-long-term stability of Zn electrode with a lifespan of up to 3500 h can be achieved at 1 mA cm^−2^. More importantly, the high Coulomb Efficiency (CE) as well as the long stability achieved in the half-cell studies of both Zn||Cu and Zn||Ti, demonstrating the good reversibility of the Zn plating/stripping process promoted by the introduction of DTPA-Na. Finally, the positive effect of DTPA-Na was further demonstrated in full cells matched with a variety of cathodes. Based on these results, the feasibility of the proposed design principle of electrolyte is verified, which can be further generalized for high-performance aqueous batteries.

## Experiments and Simulations

### Preparation of Electrolytes for Aqueous Zinc-ion Batteries

Zinc sulfate (ZnSO_4_·7H_2_O, Macklin, 99%) was dissolved into the deionized water to obtain the 2 M ZnSO_4_ baseline electrolyte. Then various concentrations of penta-sodium diethylenetriaminepentaacetic acid (C_14_H_18_N_3_Na_5_O_10_, DTPA-Na, Macklin) as the additive were added into the baseline electrolyte. The as-prepared baseline electrolyte and the improved electrolytes were used in the coin cells, H-type cells, the transparent-molds cell for in-situ optical microscopy observations, and full cells.

### Synthesis of NH_4_V_4_O_10_ Cathode

NH_4_V_4_O_10_ cathode was synthesized by a hydrothermal method [44]. Typically, 1.17 g commercial ammonium vanadate (NH_4_VO_3_, Meryer, 99%) powders were dissolved in 70 mL deionized (DI) water at room temperature. Then 1.891 g oxalic acid (H_2_C_2_O_4_·2H_2_O, Meryer, 99%) was added into the solution under vigorous stirring. Later, the mixed solution was transferred into a 100 mL Teflon-lined stainless-steel autoclave. The autoclave was heated at 140 °C for 12 h and then cooled to room temperature naturally. The obtained products were washed with deionized water for 3 times, followed by drying under vacuum at 60 °C overnight to get the final NH_4_V_4_O_10_ powders.

### Synthesis of NaV_6_O_15_ Cathode

NaV_6_O_15_ cathode was also synthesized by a hydrothermal method [45]. Typically, 727.5 mg vanadium pentoxide (V_2_O_5_, Macklin, 99%) powders and 160 mg sodium hydroxide (NaOH, Greagent, 96%) were dissolved in 70 mL of DI water. The mixed solution was transferred into a 100 mL Teflon-lined stainless-steel autoclave, which was heated at 180 °C for 24 h. After natural cooling, the products were filtered, washed, and dried.

### Characterizations

Morphology and microstructure of the samples were examined by field emission scanning electron microscopy (FE-SEM, Hitachi, S-4800, Japan) equipped with an energy-dispersive X-ray spectrometer (EDX). Fourier transformed infrared (FTIR) spectra were measured using a spectrophotometer (VERTEX 70 V) by pressed KBr pellets. Raman spectra were measured using a Raman spectrophotometer (Horiba JobinY von, HR800, France) with 633 nm laser radiation in the range of 200–2000 cm^−1^. X-ray diffraction (XRD) data were measured using a Bruker X-ray diffractometer (D8 ADVANCE A25) with Cu Ka (*λ* = 0.154178 nm) radiation. X-ray photoelectron spectroscopy (XPS) data were measured with an ESCALAB 250 Xi electron spectrometer from VG Scientific using 300 W Al Ka radiation.

### Electrochemical Measurements

The composite cathodes were prepared by the following procedure. Typically, the cathode slurry was prepared by mixing 70 wt% active material, 20 wt% carbon black, and 10 wt% polyvinylidene difluoride (PVDF) in N-methylpyrrolidone (NMP), and then coated on stainless-steel foils and dried at 60 °C in vacuum for 12 h. Both the cathode and zinc metal anode foils were punched into disks (Φ = 12 mm for coin cells and Φ = 8 mm for *H*-type cells). CR2032 coin-type cells were assembled using a glass fiber separator with a diameter of 19 mm. A fixed amount of electrolyte (60 μL for symmetric cell and half-cell, and 100 μL for full cell) was added to each coin cell. Charge/discharge tests were conducted using a Land cell test system (Land CT2001A, China). Cyclic voltammetry (CV) measurements were conducted on an electrochemical workstation (CHI614E, China) between 0.2 and 1.6 V at a sweep rate of 0.1 mV s^−1^. The electrochemical impedance (EIS) data of the cells were collected on an electrochemical workstation over a frequency range from 10^5^ to 0.1 Hz with an amplitude of 5 mV_rms_. Linear scanning voltammetry (LSV) and Tafel tests were also measured on the electrochemical workstation.

### Atomistic Simulations

Spin-polarized density functional theory (DFT) calculations were performed according to the first principles [46] within the generalized gradient approximation (GGA) using the Perdew-Burke-Ernzerhof (PBE) formulation [47]. Projected augmented wave (PAW) potentials [48] were used to describe the ionic cores and valence electrons were taken into account using a plane wave basis set with a kinetic energy cutoff of 450 eV. Van der Waals interactions have been considered using the DFT-D3 method of Grimme [49]. A geometry optimization was considered convergent when the energy change was smaller than 0.02 eV Å^−1^. During the relaxation, the Brillouin zone was sampled using a 1 × 1 × 1 Gamma centered grid for geometric optimization and transition state searching. A 15 Å-thick vacuum layer was added to the surface to eliminate the artificial interactions between periodic images.

## Results and Discussions

### Chelation Strength Design

**Fig. 1:**
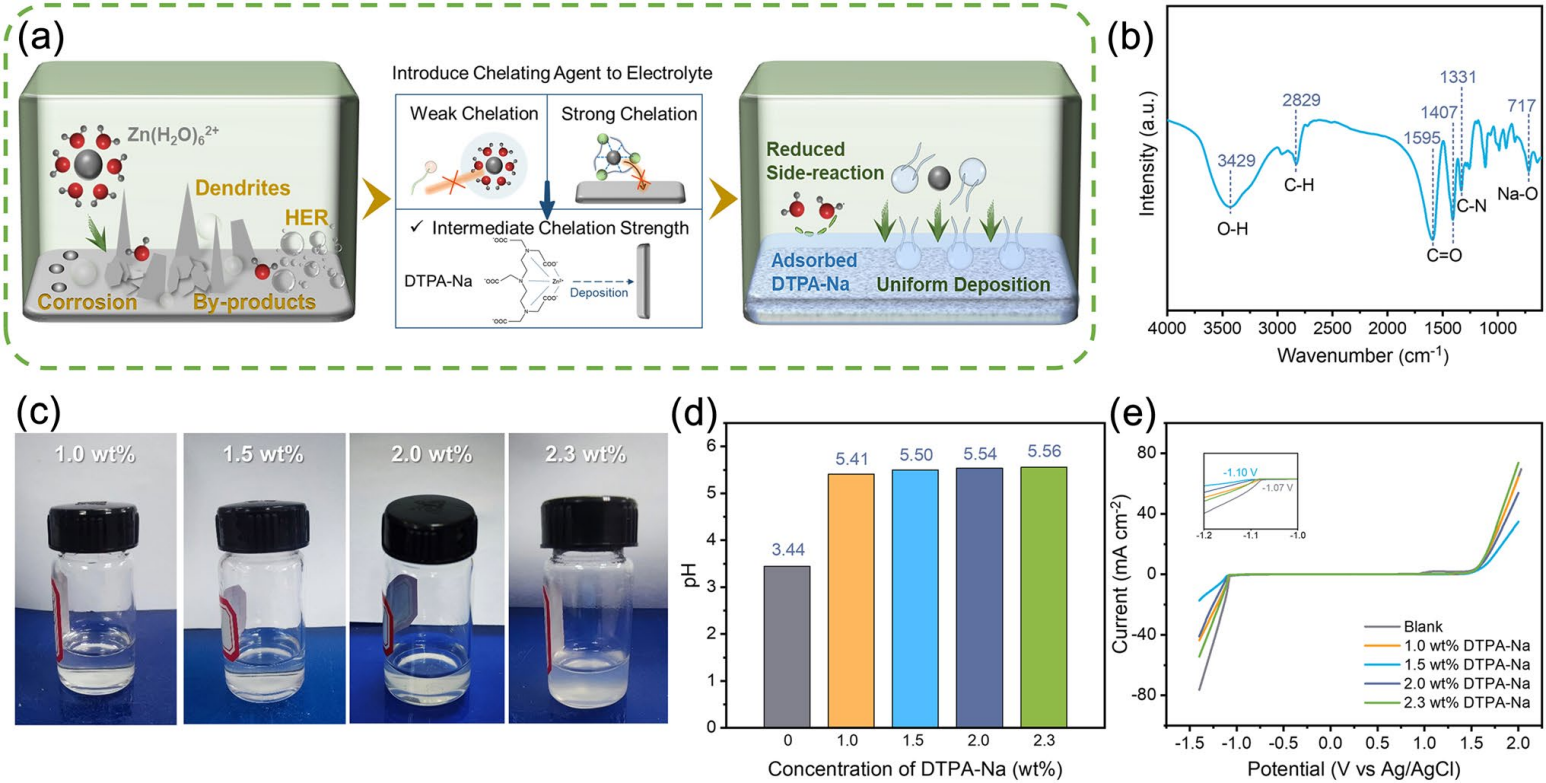
**a** Schematics showing the effect of electrolyte additives with various chelation strengths on the stability of Zn/electrolyte interface.** b** FT-IR spectra of DTPA-Na solution. **c** Digital photographs of electrolytes with different DTPA-Na concentrations.** d** pH value and** e** LSV curve of electrolytes with different DTPA-Na concentrations

The chemical and electrochemical interactions between the zinc electrode and the electrolyte are schematically shown in Fig. 1a. In the control electrolyte, 2 M ZnSO_4_ without any electrolyte additives (denoted as blank electrolyte), the uneven deposition on the surface of the zinc anode leads to the formation of dendrites. Such dendrite growth increases the surface area of zinc metal anode, which promotes HER in weakly acidic solution. As a result, HER happens as evidented by continuous bubbling during zinc plating. To solve the problem and inspired by the Sabatier principle for catalyst design [50], we proposed to use a chelating agent DTPA-Na, with intermediate chelation strength to zinc ion, to suppress HER while allowing for stable zinc stripping/plating. The stability constants of zinc ion-coordination compounds are used to reflect the stability of the chelating agent in forming complexes with zinc ions, which can serve as a reference value for the chelation strength (Table S1). The stabilization constant of DTPA-Na is 18.2 which is stronger than most chelating agents such as NTA (Nitrilotriacetic acid) and is relatively weaker than chelating agents like DOTA (1,4,7,10-tetraazacyclododecane-*N*,*N*′,*N*,*N*′-tetraacetic acid, another widely known strong chelating agent). The chelation strength of DTPA-Na is strong enough to exclude water molecules from the zinc metal-electrolyte interface (thus suppressing HER and zinc metall corrosion) and not too strong to cause a significant energy barrier for zinc ion dissociation (thus suppressing dendrite formation). The performance of the different additives will be compared later.

Since the structure of the additive plays a key role in this action, FTIR spectra were first obtained for the DTPA-Na solution. As shown in Fig. 1b, the peak at 3429 cm^−1^ is assigned to symmetric stretching vibrations of O–H. The peaks at 1595 and 1407 cm^−1^ correspond to the antisymmetric and symmetric stretching vibrations of C=O in the carboxylate, respectively. The band at 1331 cm^−1^ corresponds to the vibration of C–N, whereas the adsorption band at 717 cm^−1^ is associated with Na–O bond [51, 52]. At the same time, the FTIR characterization results of the 2 M ZnSO_4_ blank electrolyte shown in Fig. S1 revealed little signal besides the O–H alluding to water. The elemental composition of DTPA-Na was investigated by XPS (Fig. S2). The de-convoluted peaks of the C 1*s* binding energies are consistent with the C–C, C–N, and O–C=O frequencies at 284.4, 285.2, and 288.1 eV, respectively, and the deconvolution of O 1*s* spectra revealed two peaks, representing C–O (530.8 eV) and C=O (535.2 eV) [53, 54]. The abundance of oxygen-containing functional groups in the DTPA-Na leads to its strong chelating capabilities and enhances its adhesion on the anode surface [55].

To investigate the optimal additive concentration, different amounts of DTPA-Na were dissolved in 2 M ZnSO_4_ electrolyte. As shown in Fig. 1c, the solutions with 1 and 1.5 wt% DTPA-Na were homogeneous and clear, while those with 2.0 and 2.3 wt% DTPA-Na showed precipitation. The precipitates were characterized by SEM, FTIR, and XPS, attributing to the chelate product of DTPA with Zn (Figs. S3–S5).Then the pH values of the electrolytes with various DTPA-Na concentrations were tested to examine the impact of the additional DTPA-Na on the side reactions occurring at the electrode (Fig. 1d). For the blank electrolyte, the pH value of 3.44 indicates an acidic condition that would make hydrogen evolution reactions more likely and lessen the stability of the anode during the battery cycle. After the addition of DTPA-Na, the pH of the electrolyte increased due to the hydrolysis of the DTPA anions. With 1, 1.5, 2.0, and 2.3 wt% DTPA-Na added into the 2 M ZnSO_4_ electrolyte, the pH values evolved to, respectively, 5.41, 5.50, 5.54, and 5.56, gradually approaching the neutral environment, which is more unfavorable for HER reactions [56]. The current densities of hydrogen and oxygen evolution reactions on the zinc electrode were measured by linear scanning voltammetry (LSV) to demonstrate the intensity of the water splitting in various electrolytes. As shown in Fig. 1e, the addition of DTPA-Na reduced the current densities of hydrogen evolution reactions in the voltage range of − 1.0 to − 1.5 V (relative to Ag/AgCl) and oxygen evolution reactions in the range of 1.6–2.0 V, demonstrating that the additive had an impact on preventing the decomposition of water. The best effect was observed for the group with 1.5 wt% additives, whereas the current density controversially increased for the 2.0 and 2.3 wt% groups, since the precipitates produced with too high concentrations instead reduced the salt concentration and the amount of DTPA anion in solution, resulting in a weaker optimization effect. The strong adsorption role of the DTPA anions in competing with H_2_O is responsible for the ability of DTPA-Na to prevent these adverse responses. The Tafel curves were also measured with the Pt wire as the counter electrode (Fig. S7). The corrosion current of the electrode in the 1.5 wt% additive-containing electrolyte was calculated to be lower (2.465 mA cm^−2^) than in the blank electrolyte (2.973 mA cm^−2^), indicating the electrode corroded at a lower rate in the modified electrolyte, both due to the weaker acidity of the electrolyte as a result of the additive addition, and the adsorption of the DTPA anion on the electrode surface. Such weaker corrosion was beneficial to the stability of the electrode.

### Electrochemical Performance with Different DTPA-Na Concentrations

**Fig. 2:**
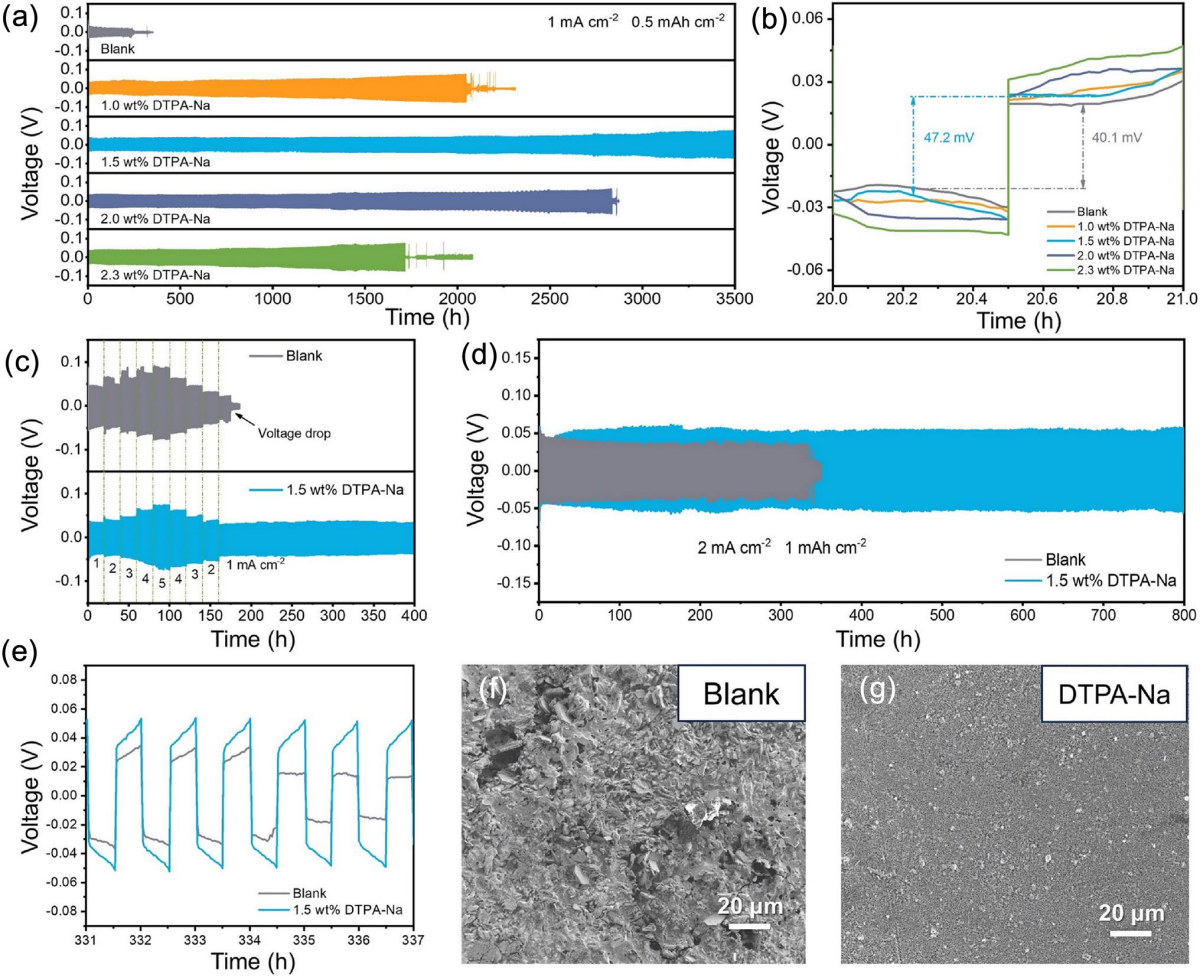
**a** Long cycle performance and **b** the related voltage–time profiles of symmetric cells assembled with different concentrations of DTPA-Na electrolyte at 1 mA cm^−2^ and 0.5 mAh cm^−2^. **c**–**d** Plating/stripping cycling stability of symmetric cells in the electrolyte at different current densities. **e** Voltage–time profiles comparison of Zn||Zn symmetric cells at 2 mA cm^−2^ and 1 mAh cm^−2^ for selected cycles. SEM images of Zn foils after 100 cycles **f** in the baseline electrolyte and **g** the designed electrolyte at 2 mA cm^−2^ with an areal capacity of 1 mA h cm.^−2^

Zn||Zn symmetric cells were assembled to evaluate the cycling stability of the cell with or without DTPA-Na additives. At a constant current of 1 mA cm^−2^ and an areal capacity of 0.5 mAh cm^−2^, as shown in Fig. 2a, the cells containing DTPA-Na exhibited more stable reversible zinc plating/stripping processes, while the cell in the blank electrolyte experienced an abrupt voltage drop at the 240th cycle, indicating the occurrence of short circuit that led to cell failure. To be more specific, the symmetrical cell with 1.0 wt% DTPA-Na in the electrolyte had improved cycling stability and was able to run for 2000 h, but the succeeding cycles encountered large voltage variations. The ultra-long cycling stability of 3500 cycles could be attained with an increase in additive content to 1.5 wt%, but further increases in additive content did not imply better cycle stability. This fact is also consistent with the LSV results, further illustrating the beneficial effect of the proper addition of DTPA-Na on improving cell performance. Figure 2b shows the voltage profile amplified during the 20th cycle. With the addition of 1.0, 1.5, 2.0, and 2.3 wt% DTPA-Na, the overpotentials of the Zn symmetric cell were found to increase sequentially from 40.1 mV for the blank electrolyte to 53.7, 47.2, 70.1, and 82.1 mV, respectively (Fig. S9). The adsorbed DTPA layer on the electrode surface results in an appropriate level of high overpotential. The solvation sheath creates a high energy barrier to cause unexpected charge transfer resistance. But the sufficient chelating agents can make it easier for the water molecules to leave the solvation sheath, resulting in the lower overpotential in the 1.5 wt% content group than in the 1.0 wt% content group. On the other hand, the larger additive content of 2.0 and 2.3 wt% will produce precipitates, which may led to higher overpotentials. Taking into account the ion conductivity and cycling stability of symmetric cells, the appropriate concentration for further investigation was chosen to be 1.5 wt%.

In order to determine the effect of the DTPA-Na additives on the Zn plating/stripping behavior, coin cells were disassembled for morphological characterizations. The SEM images of the Zn electrode after 100 cycles in the blank electrolyte demonstrated uneven Zn cluster growth, and the surface of the electrode was filled with flaky Zn deposits, corrosion pits, and by-products, which eventually led to cell failure (Fig. S10). However, the addition of 1.5 wt% DTPA-Na led to the observation of a smoother surface with grain-fine Zn deposition, which aided in the formation of a dendrite-free electrode surface. The rate performance test was carried out for the electrolytes with or without the addition of 1.5 wt% DTPA-Na. Figure 2c shows the voltage profiles of the symmetric cells at current densities ranging from 1 to 5 mA cm^−2^ for 20 h of each cycle with the current density subsequently gradually decreasing back to 1 mA cm^−2^. As current densities increased to 2, 3, 4, and 5 mA cm^−2^, the overpotentials of the cell with additives reached 40.9, 45.6, 62.3, and 73.8 mV, respectively (Fig. S11). When the current density was returned to 1 mA cm^−2^ again, it dropped to approximately 30 mV, indicating good cycling stability. Comparatively, the cell assembled with the blank electrolyte displayed instabilities in overvoltage fluctuations when the current density was changed, which may have been caused by the generation and shedding of “dead zinc” following uneven deposition/exfoliation. The subsequent overpotentials were in turn higher than those of the modified cell, originating from considerable Zn corrosion and passivated by-products. Additionally, the cell then quickly experienced a short circuit. Compared to unmodified cells, which failed after 330 cycles at 2 mA cm^−2^ and 1 mAh cm^−2^, cells containing 1.5 wt% DTPA-Na cycled steadily for a longer period of time without experiencing substantial overpotential changes (Fig. 2d). The enlarged voltage–time graph for the selected cycles provided additional evidence of the impact of the addition on the stability of the cell (Figs. 2e and S12). The modified symmetrical cell also outperformed the original one at a current density of 5 mA cm^−2^, which could run for over 450 h (Fig. S13). These results are also very competitive with the cutting-edge studies as summarized in Table S2.

In order to observe the electrode morphology more clearly and confirm the protective effect of the DTPA-Na additive, symmetrical cells were assembled using the H-shaped mold (Fig. S14). Two zinc electrode foils were sandwiched opposite each other in tubes on either side, and the backsides of the foils were covered with insulating tape to maintain a uniform effective surface. The surface morphology of the electrodes was examined by electron microscopy after 100 h of cycling at a current of 2 mA cm^−2^ with a capacity of 1 mAh cm^−2^. Due to corrosion side reactions and uneven zinc deposition, many visible protrusions and holes appeared on the surface of the zinc foil tested in the blank electrolyte (Fig. 2f). According to previous studies, the presence of the “tip effect” leads to preferential deposition of zinc on the dendrites [57, 58]. As a result, tiny protrusions gradually grow until being able to puncture the separator and eventually leading to short-circuiting of the cells. During cycling, bubbles caused by side reactions like HER are constantly present, and the growth of dendrites exposes additional sites for corrosion and side reactions. All of the above will lead to a reduction in coulombic efficiency (CE) as well as cycling performance. In sharp contrast, the zinc foil in the 1.5 wt% DTPA-Na-containing electrolyte showed a flat surface with fine grains and uniform deposition without discernible protrusions (Fig. 2g), indicating the added DTPA-Na effectively protected the zinc electrode by suppressing side reactions and uneven deposition, which further inhibits the formation of dendrites.

### Mechanisms of Stabilized Electrode/Electrolyte Interface

**Fig. 3:**
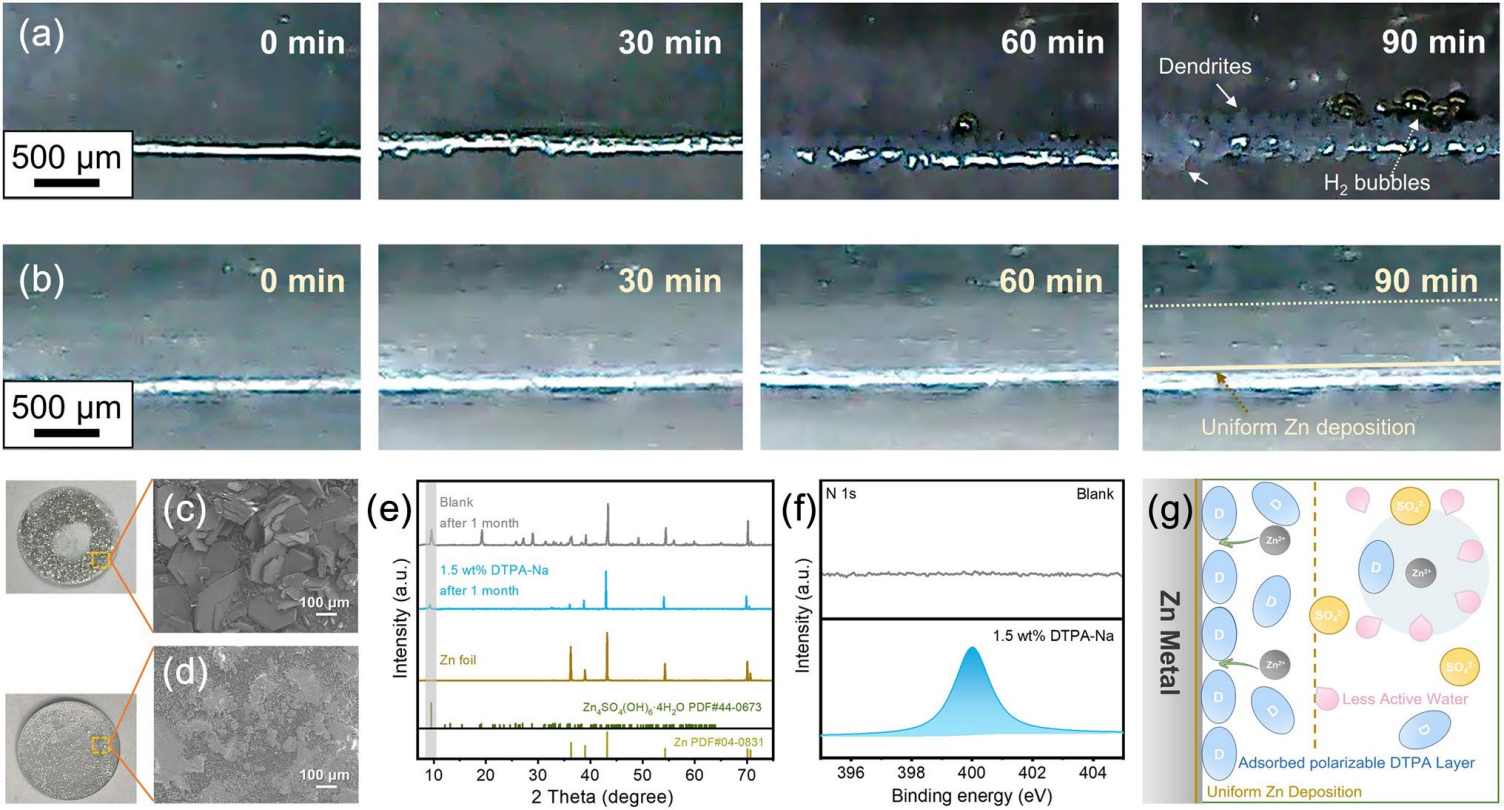
In situ optical microscopy images of the cross-sectional Zn deposition morphology **a** in the blank electrolyte and **b** the modified electrolyte in symmetrical cells at a current density of 10 mA cm^−2^. Surface morphology of Zn foils immersed in **c** the blank electrolyte and **d** the modified electrolyte for 1 month. The corresponding (**e**) XRD patterns and **f** the high-resolution XPS spectra for N1s of the immersed Zn foils. **g** Schematic illustration of the effect of DTPA on the desolvation process in the electrolytes

To further validate the mechanism of action of DTPA-Na, the plating process of the transparent symmetric cell was observed by in situ optical microscopy at a current density of 10 mA cm^−2^. For the unmodified cell, a hydrogen bubble appeared on the surface of the electrode after 60 min of plating, and the deposited zinc layer started to become uneven. As this bubble continued to grow, more dense bubbles were created one after another. Impressively, after 90 min, multiple distinct Zn dendritic crystals rapidly formed next to the bubbles (Fig. 3a). In contrast, no bubbles were detected on the electrode with the addition of 1.5 wt% DTPA-Na, and the surface of the electrode maintained a smooth morphology during the plating process (Fig. 3b). Such monitored phenomena suggested H_2_ evolution corrosion can be well mitigated by the introduction of the DTPA-Na additive. DTPA was adsorbed on the surface of Zn foil after cycling verified by Raman spectra (Fig. S15). Meanwhile, since DTPA could be readily polarized under electrical loading conditions, the concentration gradient of the electrolyte approaching the zinc electrode surface during the deposition process could also be detected in Fig. 3b. The polarizable chelate ions reached the surface of the zinc electrode with the presence of an electric field, and the complex layer located on the interface formed a dynamically regulated channel that could modify the original water clusters in the electrolyte solution. With the cooperation of the dynamic layer, the contact between water molecules and the electrode surface is somehow hindered, further improving the passivation and reducing the corrosion as well as by-products. It is worth noting that, unlike the artificial surface coatings in some research methods which are prone to structural damage after long cycles, such dynamic layer will not be affected by zinc plating/stripping but can be self-shaped and adjusted with changes in the electric field, which is more conducive to the long cycle stability of the cell.

Even at rest, the DTPA ions adsorbed on the interface also help mitigate the self-corrosion of the zinc electrode, which was investigated by immersing the Zn foil in the 2 M ZnSO_4_ electrolyte solution with/without DTPA-Na. The Zn foil in the blank electrolyte turned gray after 1 month of immersion, which was caused by severe interfacial side reactions between Zn and the electrolyte. SEM images in Fig. 3c show a large number of irregular flakes of by-products loosely accumulated on the surface of the foil, which was identified to be Zn_4_SO_4_(OH)_6_·4H_2_O (JCPDS No. 44-0673) via EDS element mapping and XRD patterns with new diffraction peaks at 9.5° (Figs. S16 and 3e). Such corrosion by-products could severely affect the ion diffusion at the interface. In contrast, the Zn foil immersed in the electrolyte containing 1.5 wt% DTPA-Na displayed little visible morphological changes as well as by-products (Fig. 3d), indicating its excellent self-corrosion protection. To further explore the dendritic inhibition mechanism of the modified electrolyte, measurements including XPS and Raman were conducted. Note that a significant N 1*s* signal was observed on the surface of the immersed Zn foil with the addition of DTPA-Na compared to that with the blank electrolyte as shown in Fig. 3f, suggesting the adsorption of DTPA-Na on the Zn foil during the immersion which is consistent with the previous observations. Also, for the C 1*s* spectrum of the Zn foil placed in the additive-containing electrolyte shown in Fig. S17, the two deconvoluted peaks were attributed to O–C=O and C–N, respectively. The abundance of oxygen-containing functional groups in the DTPA anion enhanced its adhesion to the electrode surface. The preliminary DFT computations also indicated that DTPA anion exhibits stronger adsorption energy of − 0.81 eV on the Zn surface compared to that of − 0.29 eV for free water (Fig. S18). The addition of DTPA-Na had the function of weakening the dissolution interaction between Zn^2+^ and water molecules, which can be confirmed by Raman results (Fig. S19). Compared to the original 2 M ZnSO_4_ electrolyte, the v-SO_4_^2−^ band centered at 984–986 cm^−1^ in the solution containing the additive showed a clear shoulder shift to higher frequencies [59], implying a tighter association of polymeric ions, which resulted from the DTPA-Na facilitating the stripping of water molecules from the Zn(H_2_O)_6_^2+^. Meanwhile, we selected four chelating agents, DOTA, DTPA, EDTA, and NTA, and added the same molar ratio (38.75 mM, which is equivalent to 1 wt% for DTPA) as additives in 2 M ZnSO_4_ (Fig. S20). The cycling performance of DTPA is optimal, followed by that of EDTA. Such results demonstrate that the chelating agents with stability constants around 18.2 are suitable for use in this application scenario of aqueous zinc-ion batteries.

Combining the above experimental and theoretical exploration, Fig. 3g summarized the overall mechanism of Zn dendritic inhibition with the DTPA-Na additive. During the plating/striping of Zn in the original ZnSO_4_ electrolyte, the Zn(H_2_O)_6_^2+^ approaching the interface generates many reactive H_2_O molecules, causing corrosion, hydrogen evolution, and passivation that ultimately lead to severe inhomogeneous deposition and dendritic growth, which has a serious negative impact on the Zn/Zn^2+^ reversible conversion process. However, by introducing DTPA-Na into the electrolyte, since the five carboxylated oxygens and the three nitrogens of the amine all have lone pair electrons that can be coordinated to Zn^2+^, the H_2_O molecules in the solvation sheath layer can be replaced by the chelate agents and left in the outer layer. The moderate chelating strength can also avoid a significant energy barrier to the dissociation of zinc ions. Then the DTPA anion adsorbed on the Zn surface further isolates the free water molecules from the Zn foil and thus the water-related side effects are inhibited. Meanwhile, the dynamic channels formed by polarizable DTPA-Na on the interface allow a more uniform flux of Zn^2+^ to reach the interface. Together with the electrostatic shielding effect of Na^+^, all of these contribute to the homogeneous deposition of Zn, thus allowing the cell to proceed stably over a long period of time.

### Electrochemical Performance of Half/Full Cells with Optimized DTPA-Na Concentration

Having demonstrated the effective function of the DTPA-Na additive in inhibiting Zn dendrite growth, further reversible plating and stripping measurements were next carried out to further evaluate the sustainability of the addition. Firstly, tests were conducted by assembling Zn||Ti half-cells with various concentrations of additives, from where the obtained coulombic efficiency can reveal the efficiency of the Zn plating/stripping process and is considered a significant parameter for measuring battery performance (Fig. S21 and Table S3). The Zn||Ti half-cell cycled in the 2 M ZnSO_4_ blank electrolyte exhibited an initial low CE followed by a slow ascent process. After only 40 cycles, it displayed a sudden reduction in CE, indicating an internal short circuit, whereas the average coulombic efficiency before cell failure was 92.4%. In contrast, the stable operation of the half-cell containing DTPA-Na can be extended to over 250 cycles, and the 1.5 wt% content group exhibited the highest average coulombic efficiency of 97.8%, indicating 1.5 wt% content also remains the better choice. In addition, to verify the role of precipitates, we removed the precipitates in the electrolyte with 2.3 wt% DTPA-Na, and the coulombic efficiency showed that the few precipitates did not have a significant effect on the performance of the cell (Fig. S22). Also, we further adjusted the pH of the electrolyte to the same value of 5.5 as that of the DTPA-Na additive with NaOH and compared the performance, and the results of the symmetric cell and the half-cell showed that the performance of the battery was not improved with the addition of NaOH, which also proved that in addition to adjusting the pH, the additive's role is more important in the chelating ability (Figs. S23 and S24).

**Fig. 4:**
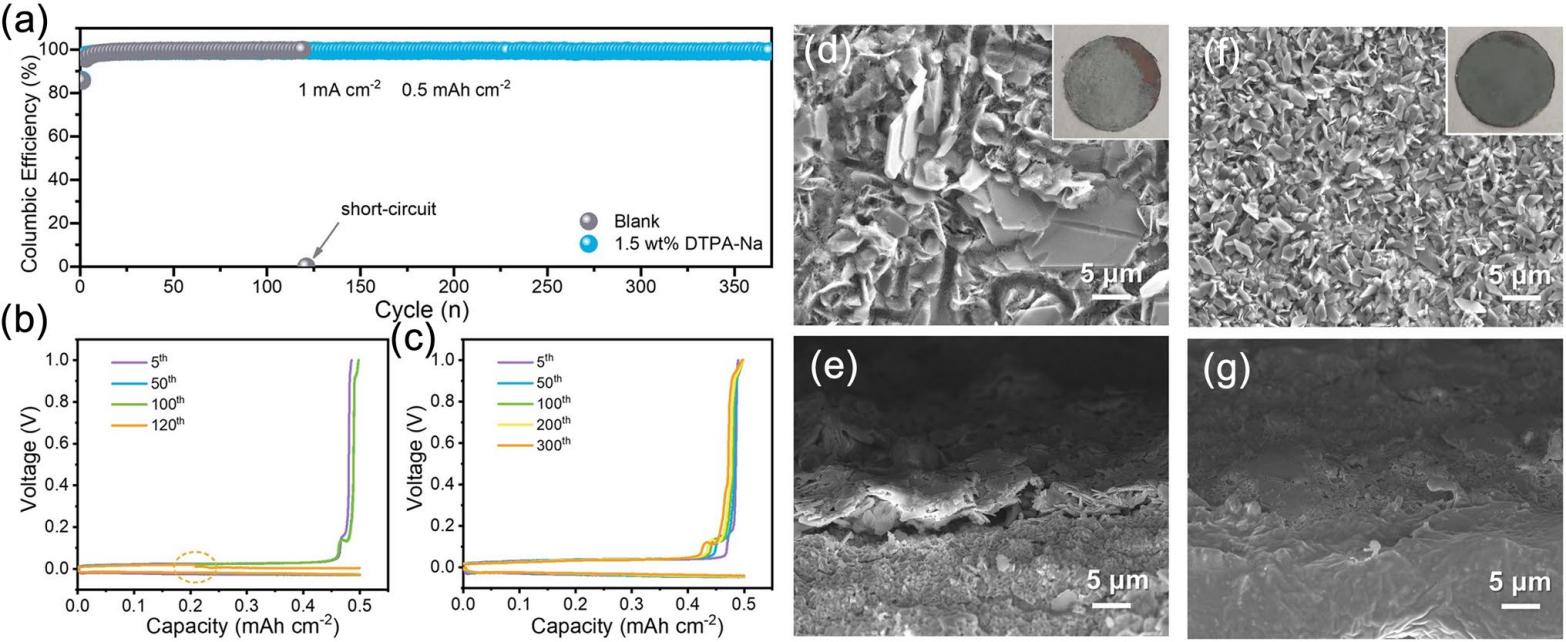
**a** Coulombic efficiency of Zn plating/stripping on Cu in the baseline and designed electrolytes. Corresponding voltage profiles of the Zn||Cu cells in **b** the baseline electrolyte and **c** the designed electrolyte at different cycles. Surface and cross-section morphology of Cu foils after 100 cycles in **d**–**e** the blank electrolyte and **f**–**g** the designed electrolyte

Then Cu foil was used as a counter electrode with a cut-off voltage of 1.0 V (vs. Zn/Zn^2+^) as shown in Fig. 4a. Zn||Cu assembled with the blank electrolyte encountered a sudden drop in CE values after 120 cycles due to dendrite growth or other side reactions. But for Zn||Cu cells using electrolytes containing 1.5 wt% DTPA-Na additive, high and stable CE values were maintained for more than 350 cycles. The voltage curve of the Zn||Cu half-cell in the blank electrolyte zigzagged down at the 120th cycle (Fig. 4b), which corresponds to the abnormal short circuit of the cell. In the meantime, the modified cell has a high overlap of the voltage curve for 300 cycles (Fig. 4c). Another set of Zn||Cu half-cells was disassembled after 100 cycles to observe the morphology of the deposited Zn on the Cu foils. For the Cu foil cycled in the blank electrolyte, the deposited Zn was in large, disorganized sheets, where the deposited surface was uneven to the naked eye (inset), and the cross-sectional view showed that the deposited Zn was poorly bonded to the base. As shown in Fig. 4d, e, the unrestricted and arbitrary plating and stripping process lead to the accumulation of "dead zinc" during repeated cycles that dramatically weakened the stability of the cell [60]. For the modified cell, the zinc grains deposited on the Cu foil with 1.5 wt% DTPA-Na in the electrolyte were small and uniform (Fig. 4f, g). These results are consistent with the long-lasting and high values of CE, again verifying that cells using DTPA-Na-containing electrolytes could exhibit better cycling stability.

**Fig. 5:**
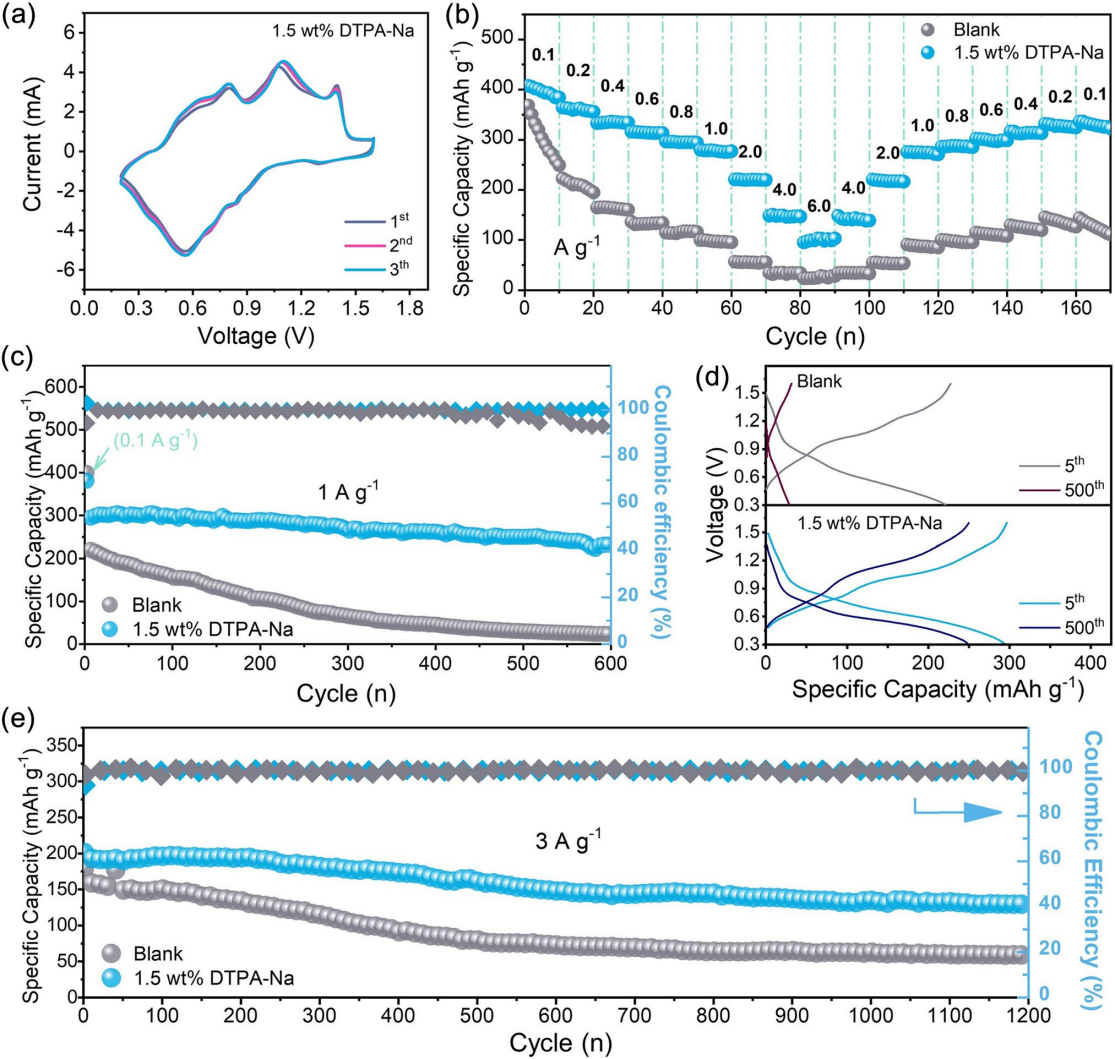
**a** CV profiles of Zn||NH_4_V_4_O_10_ cell in the designed electrolyte at 0.1 mV s^−1^. **b** Rate capability from 0.1 to 6 A g^−1^. **c** Cycling performance at 1 A g^−1^ in the voltage range of 0.2–1.6 V (vs Zn/Zn^2+^) and **d** corresponding discharge/charge profiles for selected cycles. **e** Long-span cycling performance of Zn||NH_4_V_4_O_10_ cells at 3 A g^−1^

Considering the outstanding electrochemical performance of the additive DTPA-Na in both the symmetric cells and the half-cells, the full zinc-ion cells coupled with the NH_4_V_4_O_10_ cathodes were assembled and tested further. The cathode active material was prepared according to the previous literature that was verified to match with the NH_4_V_4_O_10_ phase (JCPDS. No. 24-1155) (Figs. S18 and S19). Figure 5a presents the first three CV curves for Zn||NH_4_V_4_O_10_ with 1.5 wt% DTPA-Na-added electrolyte within the potential window of 0.2–1.6 V (vs. Zn^2+^/Zn) at a scan rate of 0.1 mV s^−1^, where the high overlapping shapes of the cycles showed the reversibility of the reaction. The rate performance of Zn||NH_4_V_4_O_10_ devices with the additive present effective enhancement compared to that of the original 2 M ZnSO_4_ electrolyte. To be specific, the modified full cell exhibited impressive discharge specific capacities of 405.4, 364.1, 332.9, 316.0, 296.3, 279.7, 220.8, and 148.5 mAh g^−1^ at 0.1, 0.2, 0.4, 0.6, 0.8, 1.0, 2.0, and 4.0, respectively, all of which are higher than that of the blank electrolyte (Figs. 5b and S20). It is worth noting that the cell with 1.5 wt% DTPA-Na could reversibly release 103.2 mAh g^−1^ even at an ultra-high current density of 6 A g^−1^, while the discharge capacity of the original cell is only 24.0 mAh g^−1^ under the same conditions, which proved the superior kinetic properties of the modified electrolytes. After high-rate discharge/charge, the average capacity of the modified cell can still recover rapidly and tend to be around 336 mAh g^−1^ when the current density drops to 0.1 A g^−1^ again, demonstrating its superior Zn^2+^ plating/stripping reversibility. The cycling stability was evaluated at the current density of 1 A g^−1^ (0.1 A g^−1^ for the first three cycles), as displayed in Fig. 5c. The capacity of the full cell assembling with the blank electrolyte decreases rapidly during the cycling, with capacity retention already below 50% (vs. the 4th cycle) after 200 cycles, and only remaining with a negligible discharge capacity of 30 mAh g^−1^ after 500 cycles, which is mainly contributed by the capacitance. Simply by adding 1.5 wt% DTPA-Na to the electrolyte, however, the Zn||NH_4_V_4_O_10_ full cell could still exhibit 249.9 mAh g^−1^ capacity after 500 cycles, corresponding to 84.6% capacity retention (Fig. 5d). Moreover, the Zn||NH_4_V_4_O_10_ cell also delivers excellent long-span cycling stability at 3 A g^−1^ (Fig. 5e). Such long-term cycling stability once again confirms the effectiveness of the additive. Another Na-containing cathode, NaV_6_O_15_, (Figs. S21 and S22) was also introduced as the cathode for comparison, and the full cell performance still demonstrated superior cycling performance over the original 2 M ZnSO_4_ electrolyte due to the effectiveness of the DTPA anion and the presence of Na^+^ contained in the additive analyzed previously, as shown in Fig. S23. These findings demonstrate once more that the DTPA-Na additive can achieve high cycle reversibility by reducing both dendrite-growth and unanticipated side effects.

## Conclusions

In summary, we proposed to select effetive electrolyte additives for AZIBs using intermediate chelation strength. The selected DTPA-Na additive can effectively modify the electrolyte for dendrite-free and highly reversible AZIBs. By dynamically modulating the anode/electrolyte interface and tuning the solvation sheath of zinc ions, the electrolyte with DTPA-Na suppresses HER and zinc-metal corrosion and regulates Zn^2+^ diffusion and deposition, leading to a highly reversible Zn anode. Over 3500 h of steady operation of the Zn||Zn symmetric cells can be achieved with stable overpotential at moderate current densities (1 mA cm^−2^ with 0.5 mAh cm^−2)^. Stable Zn plating/stripping processes on Cu foils can be obtained for > 500 cycles with stable CE close to 100%. When applied in Zn||NH_4_V_4_O_10_ full cells, it enables a high capacity retention of 84.6% after 500 cycles. This work opens a new door for addressing the corrosion and dendrite problems in AZIBs and offers a practical approach to the logical design of reliable aqueous electrolytes.

## Supplementary Information

Below is the link to the electronic supplementary material.

Supplementary file1 (PDF 2057 KB)
