# Supplementary material for: Polarizable Additive with Intermediate Chelation Strength for Stable Aqueous Zinc-Ion Batteries
Source: Nanomicro Lett. 2024 Jan 12;16:82. doi: 10.1007/s40820-023-01305-0 (PMC10786796; doi:10.1007/s40820-023-01305-0)
Supplement: Supplementary file 1 — Supplementary file1 (PDF 2057 KB) [file 40820_2023_1305_MOESM1_ESM.pdf]

Supporting Information for

Polarizable Additive with Intermediate Chelation Strength for Stable Aqueous Zinc-Ion Batteries

Yuting Xia[1], Rongao Tong[1], Jingxi Zhang[1], Mingjie Xu[2], Gang Shao[2], Hailong Wang[2], Yanhao Dong[1,*], Chang-An Wang[1,*]

[1]State Key Lab of New Ceramics and Fine Processing, School of Materials Science and Engineering, Tsinghua University, Beijing 100084, People's Republic of China

[2]School of Materials Science and Engineering, Zhengzhou University, Zhengzhou, Henan 450001, People's Republic of China

*Corresponding authors. E-mail: dongyanhao@tsinghua.edu.cn (Yanhao Dong); wangca@tsinghua.edu.cn (Chang-An Wang)

Supplementary Figures and Tables

Table S1 Stability Constants of Zinc ion-Coordination Compounds (25 °C, the number of ligand is 1) [S1]

**Ligand**	**lgβ**
DOTA	19.7
DTPA	18.2
EDTA	16.3
NTA	10.7
Lactic acid	6.85
Oxalic acid	1.5

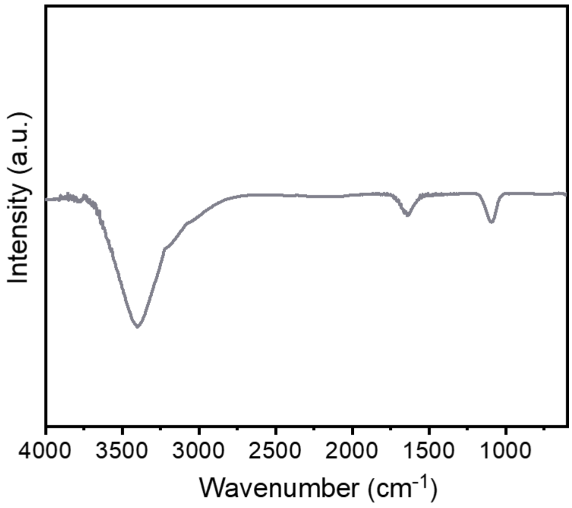

Fig. S1 FT-IR spectra of 2M $ZnSO_4$ electrolyte

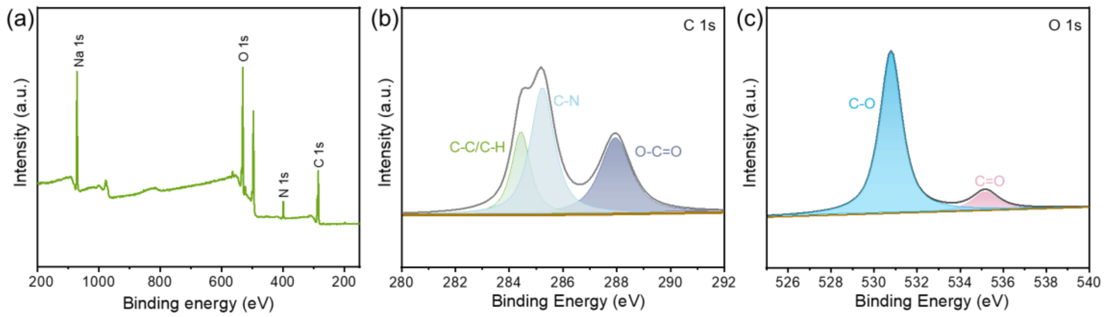

Fig. S2 XPS spectra of the DTPA-Na powder: **a** survey scan, **b** C 1s and **c** O 1s

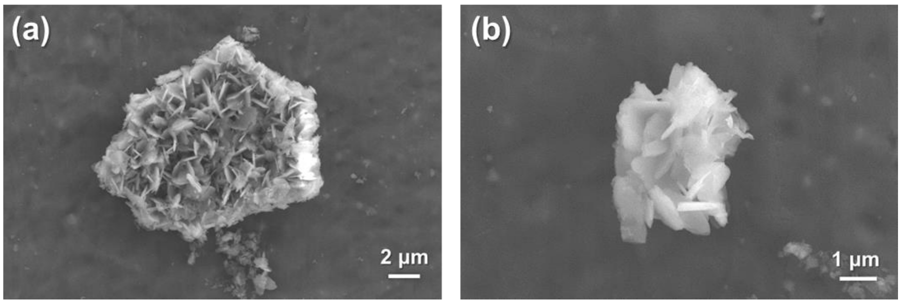

Fig. S3 SEM images of the precipitates in the electrolyte with 2.3 wt% DTPA-Na

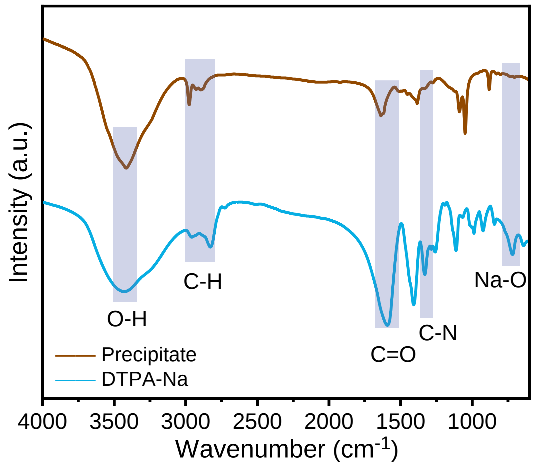

Fig. S4 FT-IR spectra of the precipitates in the electrolyte with 2.3 wt% DTPA-Na

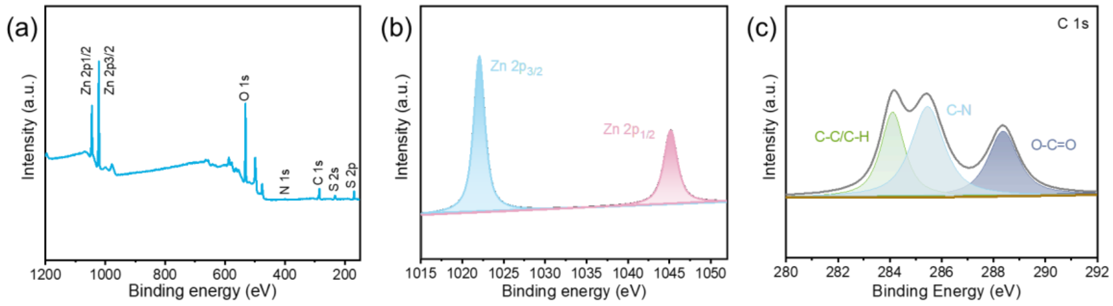

Fig. S5 XPS spectra of the precipitates: **a** survey scan, **b** Zn 2p, **c** C 1s

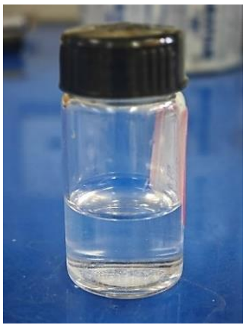

Fig. S6 Digital photographs of the precipitates in the electrolyte with 2.3 wt% DTPA-Na

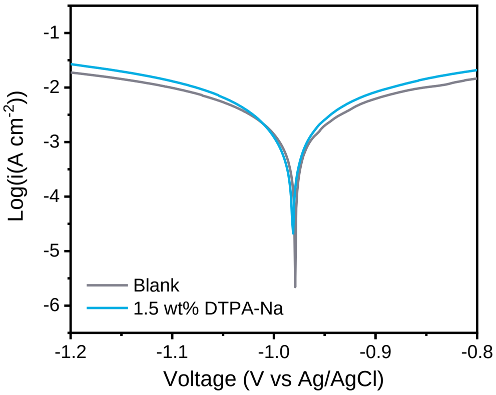

Fig. S7 Linear polarization curves showing the corrosion on Zn foils

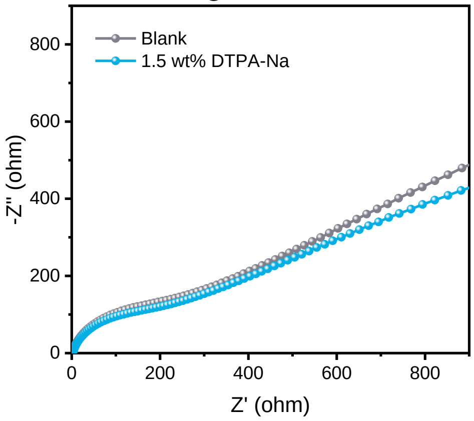

Fig. S8 Nyquist plots of the Zn||Zn symmetric cells in the electrolytes with and without DTPA-Na addition

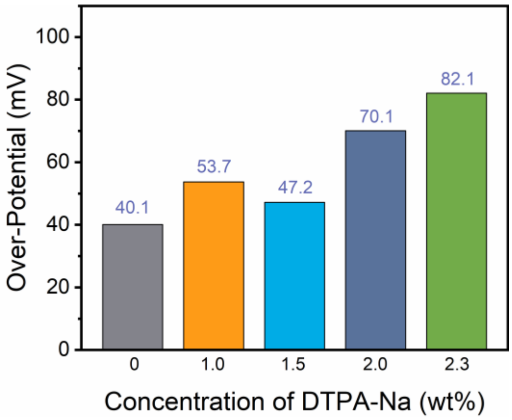

Fig. S9 Over-potentials of symmetric cells during the 20th cycle with different concentrations of DTPA-Na in the electrolyte

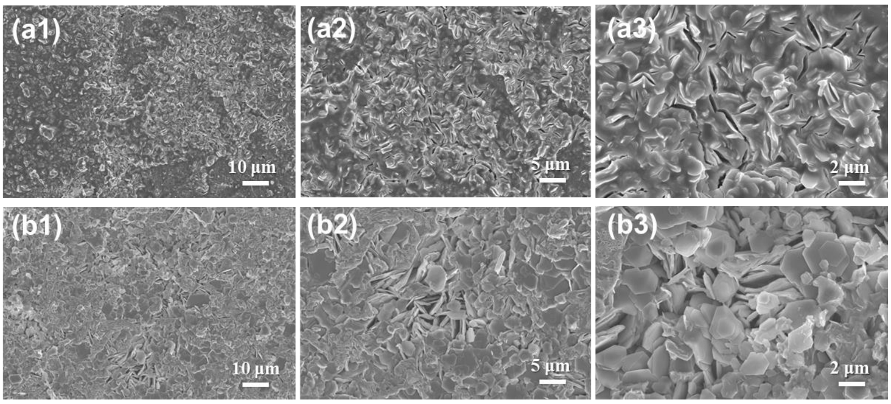

Fig. S10 Surface morphology of Zn foils after 100 cycles in Zn||Zn symmetric coin cells with **a** the blank electrolyte and **b** the designed electrolyte

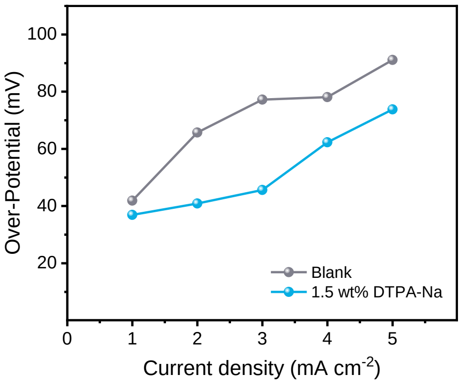

Fig. S11 Over-potentials of Zn||Zn symmetric cells at current densities from 1 to 5 mA cm^{-1}

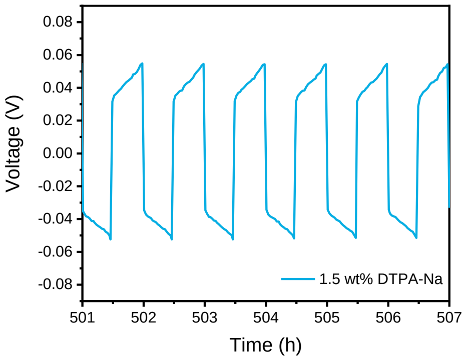

Fig. S12 Voltage-time profiles comparison of Zn||Zn symmetric cells at 2 mA cm^{-2} and 1 mAh cm^{-2} for the selected cycles

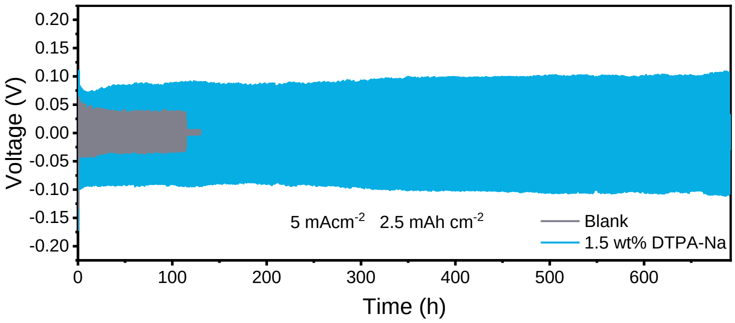

Fig. S13 Long cycle performance of symmetric cells at 5 mA cm^{-2} and 2.5 mAh cm^{-2}

Table S2 Comparisons of cycling performance of the designed electrolytes in symmetrical batteries with some reported modified anodes for AZIBs

No.	Electrolyte Component	Current Density (mA cm^{-2})	Areal Capacity (mAh cm^{-2})	Cycling Duration (h)	References
1	2 M $ZnSO_4$ + 1.5 wt% DTPA-Na	1<br>2<br>5	0.5<br>1<br>2.5	3500<br>800<br>450	**This work**
2	2 M $ZnSO_4$ + 0.0085 m $La(NO_3)_3$	1	1	1200	[S2]
3	2 M $ZnSO_4$ + 75 × 10^{-3} m Na_4EDTA	2<br>5	2<br>2	450<br>2000	[S3]
4	2 M $ZnSO_4$ + 8 mg mL^{-1} PASP	0.5<br>20	0.5<br>1	3200<br>200	[S4]
5	2 M $ZnSO_4$ + 1 vol% DM	2	2	400	[S5]
6	2 M $ZnSO_4$ + 0.2 M $MnSO_4$ + 1% Et_2O + 30% EG	0.5<br>5	0.5<br>5	700<br>700	[S6]
7	2 M $ZnSO_4$ + 0.5 g L^{-1} TMBAC	2<br>10	2<br>5	900<br>470	[S7]
8	2 M $ZnSO_4$ + 0.5 g L^{-1} Sac	10	10	600	[S8]
9	1 M $ZnSO_4$ + 5% 1,4-Dioxane	5	2.5	600	[S9]
10	1 M $ZnSO_4$ + 10 vol% PG	2	2	1000	[S10]
11	3 m $ZnAc_2$ + 30 m KAc + 3 m LiAc	0.1	0.2	1000	[S11]
12	4 M $Zn(CF_3SO_3)_2$ + 0.5 M $Me_3EtNOTF$	0.5	0.25	6000	[S12]
13	1.3 m $ZnCl_2/H_2O$–DMSO (volume ratio of H_2O/DMSO = 4.3:1)	0.5	0.5	1000	[S13]
14	30 m $ZnCl_2$	0.2	0.03	600	[S14]
15	3 m $Zn(OTf)_2$ + 17 m $NaClO_4$	1	0.25	1600	[S15]

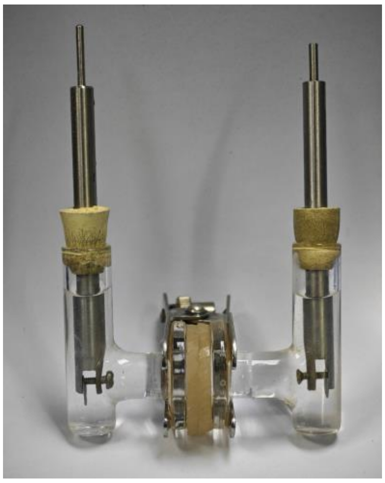

Fig. S14 Symmetrical cells assembled in transparent H-type tanks

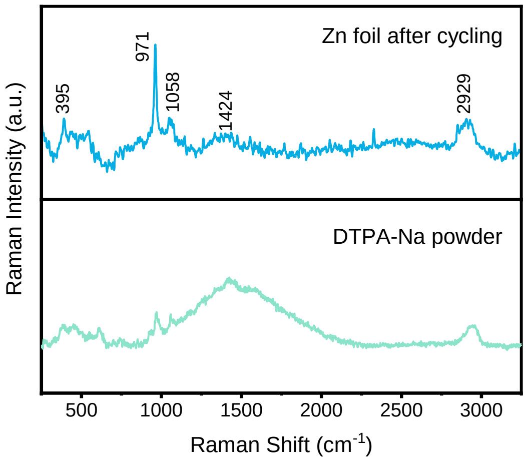

Fig. S15 Raman spectroscopies of the surface of the Zn foils after 100 cycles in Zn||Zn symmetric coin cells with the designed electrolyte

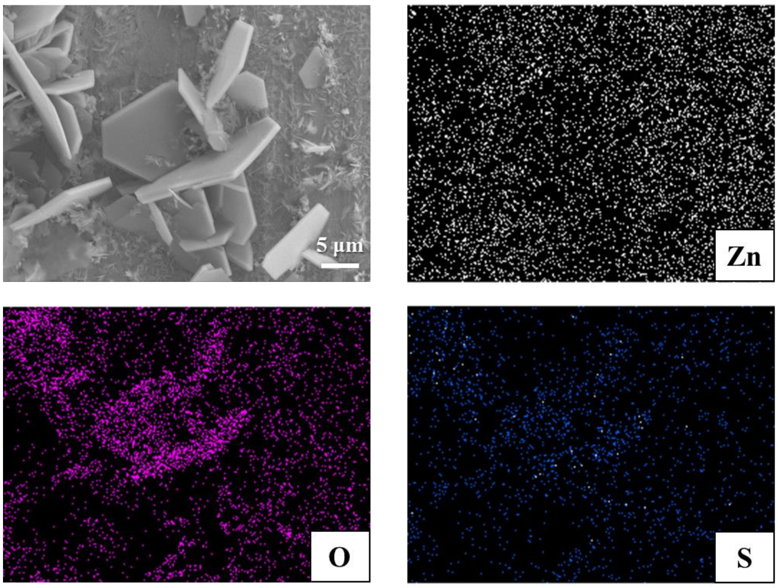

Fig. S16 Surface morphology and corresponding EDS element mapping of Zn, O, and S of the Zn foil immersed in 2M $ZnSO_4$ electrolyte for one month

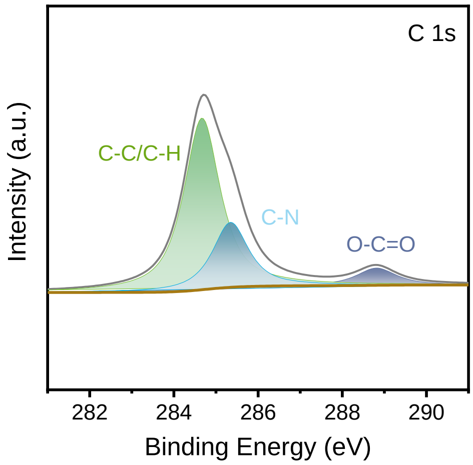

Fig. S17 XPS high-resolution for C 1s of the Zn foil after immersed in the electrolyte with 1.5 wt% DTPA-Na for one month

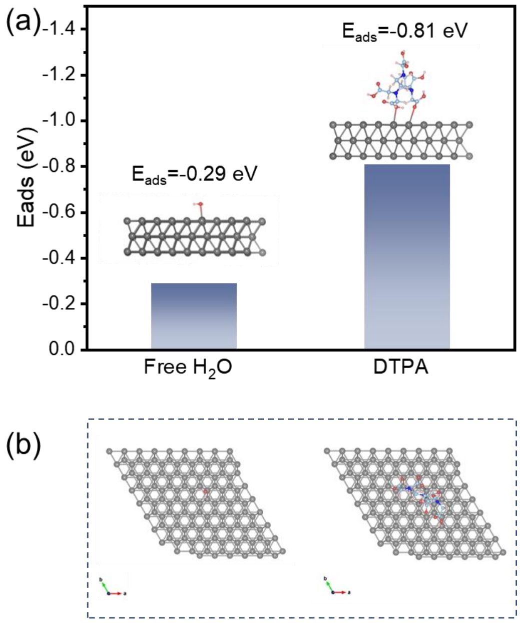

Fig. S18 Adsorbed models of free H_2O and DTPA on Zn (002) surface and corresponding adsorption energy

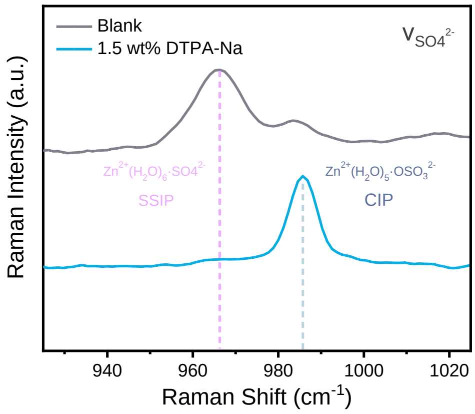

Fig. S19 Raman spectroscopies of the surface of the Zn foils after immersed in different electrolytes for one month

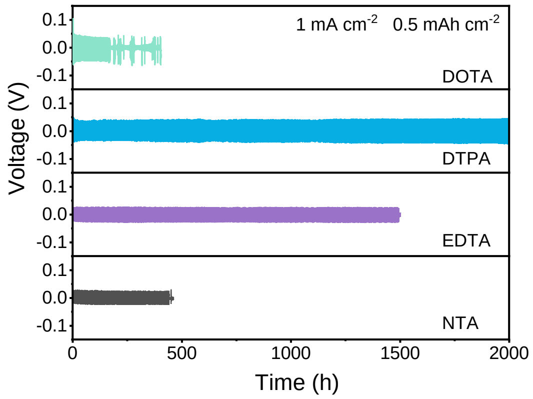

Fig. S20 Long cycle performance of symmetric cells assembled with different ligands at 1 mA cm^{-2} and 0.5 mAh cm^{-2}

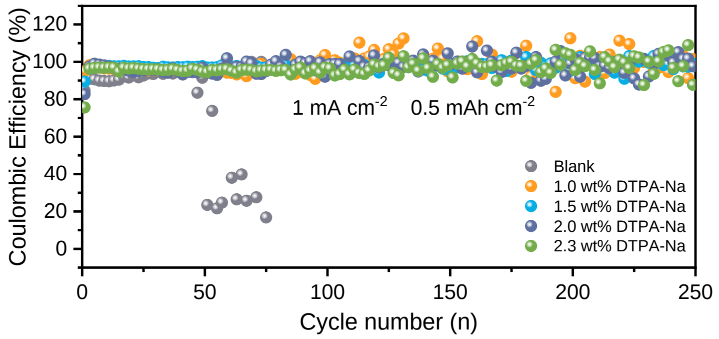

Fig. S21 Coulombic efficiency of Zn plating/stripping on Ti foils in the different electrolytes at 1 mA cm^{-2} with 0.5 mAh cm^{-2}

Table S3 The average CE of Zn||Ti half cells in the electrolytes with different concentrations of DTPA-Na after 250 cycles

Electrolyte component	Average CE (%)
Blank	92.4 (40 cycles)
1.0 wt% DTPA-Na	97.4
**1.5 wt% DTPA-Na**	**97.8**
2.0 wt% DTPA-Na	97.4
2.3 wt% DTPA-Na	96.9

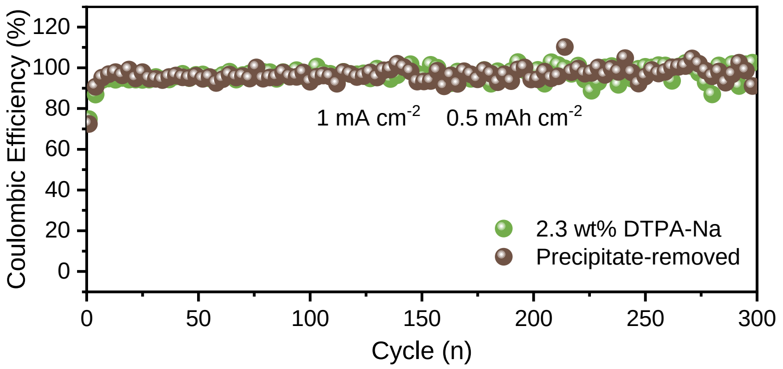

Fig. S22 Coulombic efficiency of the Zn||Ti half cell with/without precipitates in electrolyte

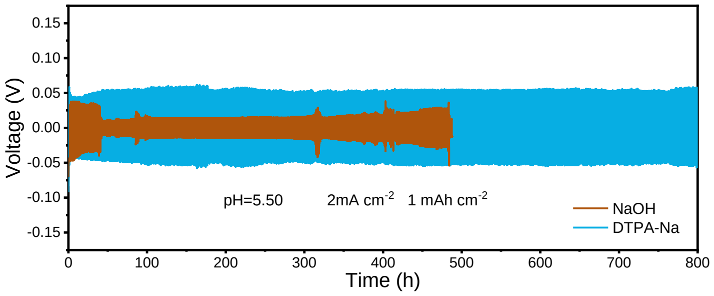

Fig. S23 Long cycle performance of symmetric cells in the electrolytes with pH of 5.50

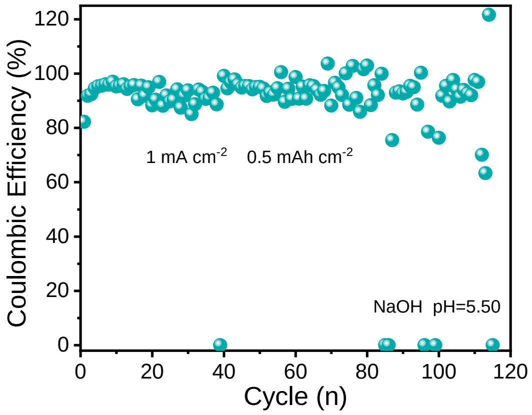

Fig. S24 Coulombic efficiency of Zn plating/stripping on Ti foil in the electrolyte with pH of 5.50 adjusted by NaOH

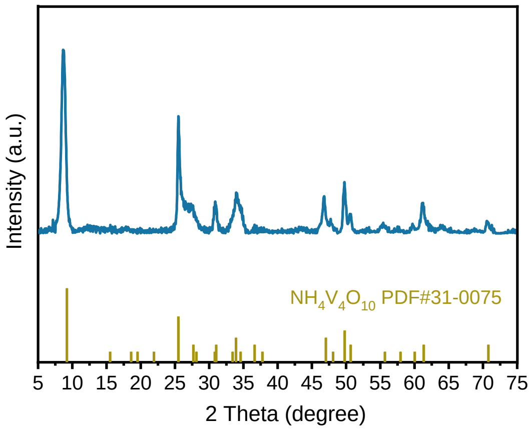

Fig. S25 XRD pattern of the prepared $NH_4V_4O_{10}$ cathode active materials

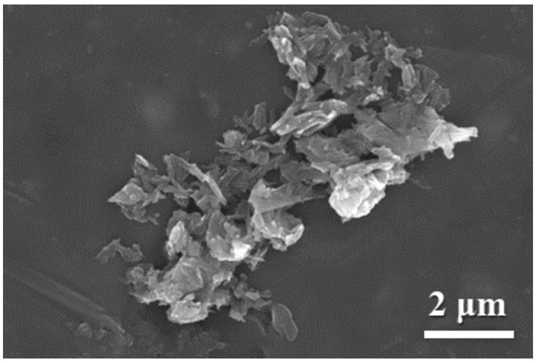

Fig. S26 SEM image of the prepared $NH_4V_4O_{10}$ cathode active materials

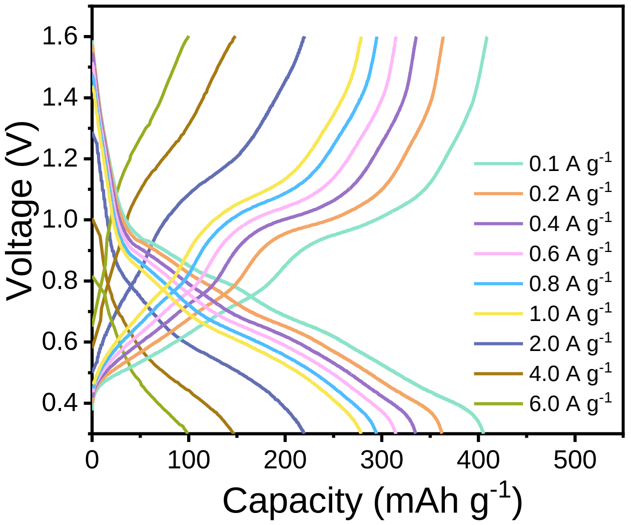

Fig. S27 Discharge/charge profiles of Zn||$NH_4V_4O_{10}$ cells at different current density

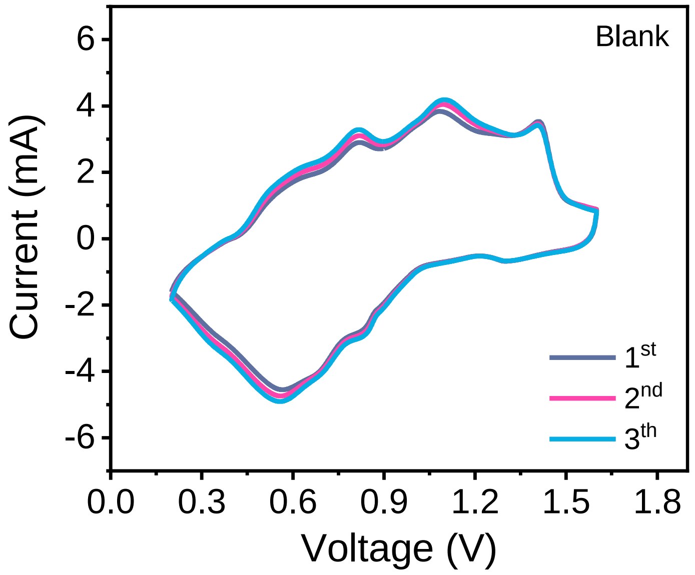

Fig. S28 CV profiles of Zn||$NH_4V_4O_{10}$ cell in the baseline electrolyte at 0.1 mV s^{-1}

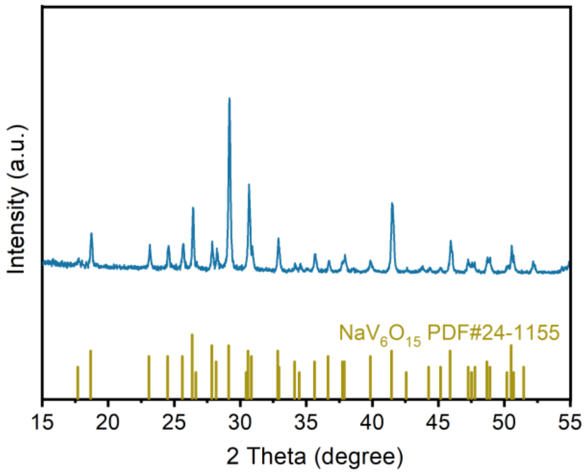

Fig. S29 XRD pattern of the prepared NaV_6O_{15} cathode active materials

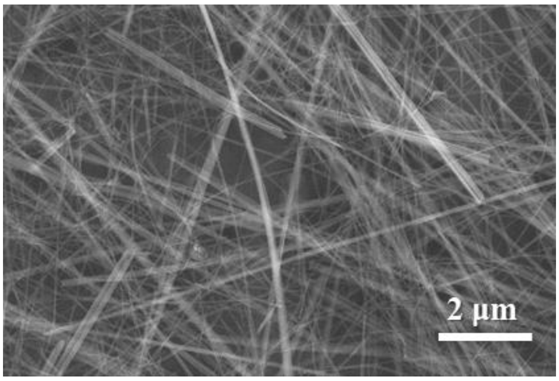

Fig. S30 SEM image of the prepared NaV_6O_{15} cathode active materials

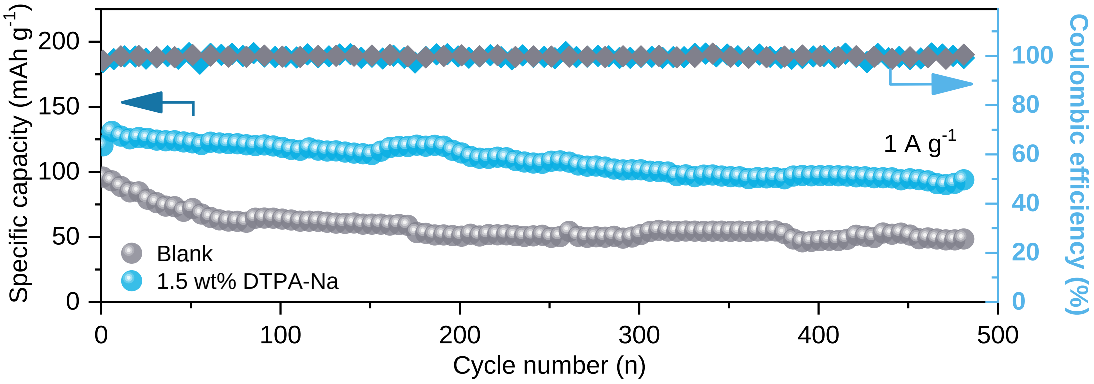

Fig. S31 Cycle performance Zn||NaV_6O_{15} cells at 1 A g^{-1}

Supplementary References

[S1] NIST standard reference database 46. http://www.nist.gov/srd/nist46.cfm

[S2] R. Zhao, H. Wang, H. Du, Y. Yang, Z. Gao et al., Lanthanum nitrate as aqueous electrolyte additive for favourable zinc metal electrodeposition. Nat. Commun. **13**, 3252 (2022). https://doi.org/10.1038/s41467-022-30939-8

[S3] S.-J. Zhang, J. Hao, D. Luo, P.-F. Zhang, B. Zhang et al., Dual-function electrolyte additive for highly reversible Zn anode. Adv. Energy Mater. **11**, 2102010 (2021). https://doi.org/10.1002/aenm.202102010

[S4] T. Zhou, Y. Mu, L. Chen, D. Li, W. Liu et al., Toward stable zinc aqueous rechargeable batteries by anode morphology modulation via polyaspartic acid additive. Energy Storage Mater. **45**, 777–785 (2022). https://doi.org/10.1016/j.ensm.2021.12.028

[S5] J. Cui, X. Liu, Y. Xie, K. Wu, Y. Wang et al., Improved electrochemical reversibility of Zn plating/stripping: a promising approach to suppress water-induced issues through the formation of H-bonding. Mater. Today Energy **18**, 100563 (2020). https://doi.org/10.1016/j.mtener.2020.100563

[S6] A. Wang, W. Zhou, A. Huang, M. Chen, Q. Tian et al., Developing improved electrolytes for aqueous zinc-ion batteries to achieve excellent cyclability and

antifreezing ability. J. Colloid Interface Sci. **586**, 362–370 (2021). https://doi.org/10.1016/j.jcis.2020.10.099

[S7] K. Guan, L. Tao, R. Yang, H. Zhang, N. Wang et al., Anti-corrosion for reversible zinc anode via a hydrophobic interface in aqueous zinc batteries (adv. energy mater. 9/2022). Adv. Energy Mater. **12**, 2270037 (2022). https://doi.org/10.1002/aenm.202270037

[S8] C. Huang, X. Zhao, S. Liu, Y. Hao, Q. Tang et al., Stabilizing zinc anodes by regulating the electrical double layer with saccharin anions. Adv. Mater. **33**, e2100445 (2021). https://doi.org/10.1002/adma.202100445

[S9] R. Feng, X. Chi, Q. Qiu, J. Wu, J. Huang et al., Cyclic ether-water hybrid electrolyte-guided dendrite-free lamellar zinc deposition by tuning the solvation structure for high-performance aqueous zinc-ion batteries. ACS Appl. Mater. Interfaces **13**, 40638–40647 (2021). https://doi.org/10.1021/acsami.1c11106

[S10] Y. Shang, P. Kumar, T. Musso, U. Mittal, Q. Du et al., Long-life Zn anode enabled by low volume concentration of a benign electrolyte additive. Adv. Funct. Mater. **32**, 2200606 (2022). https://doi.org/10.1002/adfm.202200606

[S11] J. Han, A. Mariani, A. Varzi, S. Passerini, Green and low-cost acetate-based electrolytes for the highly reversible zinc anode. J. Power Sources **485**, 229329 (2021). https://doi.org/10.1016/j.jpowsour.2020.229329

[S12] L. Cao, D. Li, T. Pollard, T. Deng, B. Zhang et al., Fluorinated interphase enables reversible aqueous zinc battery chemistries. Nat. Nanotechnol. **16**, 902–910 (2021). https://doi.org/10.1038/s41565-021-00905-4

[S13] L. Cao, D. Li, E. Hu, J. Xu, T. Deng et al., Solvation structure design for aqueous Zn metal batteries. J. Am. Chem. Soc. **142**, 21404–21409 (2020). https://doi.org/10.1021/jacs.0c09794

[S14] C. Zhang, J. Holoubek, X. Wu, A. Daniyar, L. Zhu et al., A $ZnCl_2$ water-in-salt electrolyte for a reversible Zn metal anode. Chem. Commun. **54**, 14097–14099 (2018). https://doi.org/10.1039/C8CC07730D

[S15] H. Ao, W. Zhu, M. Liu, W. Zhang, Z. Hou et al., High-voltage and super-stable aqueous sodium-zinc hybrid ion batteries enabled by double solvation structures in concentrated electrolyte. Small Methods **5**, e2100418 (2021). https://doi.org/10.1002/smtd.202100418
